# Longitudinal proteomics study of serum changes after allogeneic HSCT reveals potential markers of metabolic complications related to aGvHD

**DOI:** 10.1038/s41598-022-18221-9

**Published:** 2022-08-17

**Authors:** Sing Ying Wong, Seiko Kato, Frans Rodenburg, Arinobu Tojo, Nobuhiro Hayashi

**Affiliations:** 1 School of Life Science and Technology, Tokyo Institute of Technology, 2-12-1 Ookayama, Meguro-ku, Tokyo, 152-8550 Japan; 2 Department of Hematology/Oncology, The Institute of Medical Science, The University of Tokyo, 4-6-1 Shirokanedai, Minato City, Tokyo, 108-8639 Japan; 3 Institute of Biology, Leiden University, Sylviusweg 72, 2333 BE Leiden, The Netherlands

**Keywords:** Biomarkers, Proteomics, Mass spectrometry, Proteomic analysis, Skin manifestations

## Abstract

Even though hematopoietic stem cell transplantation (HSCT) allows successful treatment for many malignant and non-malignant disorders, its curative potential remains limited by severe side effects, including infections and other transplant-related complications such as graft-versus-host disease (GvHD). This study examined changes in serum proteome via high-performance two-dimensional gel electrophoresis (2-DE) during HSCT to search for diagnostic biomarkers for post-HSCT complications. Longitudinal proteomic analysis revealed proteins related to metabolic complications and hemolytic anemia. Retinol-binding protein 4 (RBP4), a reliable marker of insulin resistance, was identified, and is possibly associated with the onset mechanism of acute graft-versus-host disease (aGvHD) and/or skin GvHD. Although the cause of insulin resistance is not fully understood, it is thought to be associated with adipocytes inflammation induced by RBP4, iron overload and hemolytic anemia after HSCT, as observed in this study. The present study has demonstrated that insulin resistance and metabolic complications could be immediate complications after transplantation and are associated with aGvHD. The biomarkers revealed in this study are promising tools to be used for improving the early diagnosis of HSCT-associated complications, especially aGvHD, possibly even before clinical manifestations.

## Introduction

HSCT is an essential immunotherapy modality, for a wide range of malignant and non-malignant disorders through the infusion of multipotent hematopoietic progenitor cells^1,2^. In allogeneic transplantation, healthy stem cells from bone marrow, peripheral blood or umbilical cord blood of donors are used as source to rebuild hematopoietic function in patients.

A range of modifications and advances in transplantation techniques have constantly improved over the years, leading to increasing numbers of long-term survivors, usually for at least 2 years^3,4^. Nonetheless, the curative potential of this treatment remains largely limited by severe side effects, including infections, recurrence of the primary disease and other transplant-related complications, including graft-versus-host disease (GvHD)^5^. Patients become highly susceptible to infections post-HSCT due to the alterations of their immune system. Moreover, there is a growing concern on the immunosuppression regimens used to prevent occurrence of transplantation complications after HSCT, such as graft rejection, which would further weaken the patient’s immune system causing increased risk of infection^6^.

Immediate survival of HSCT recipients is thus not the sole concern anymore. Measures to reduce the severity of side effects, such as GvHD, are urgently needed. Approximately 40–60% of HSCT recipients experience acute GvHD (aGvHD) despite immunosuppressive prophylaxis^7,8^. Acute GvHD occurs when the donor T lymphocytes recognise the host’s immune cells as foreign and attack them^9^. The activated pathogenic T cells would then infiltrate target organs such as the skin, liver and gastrointestinal (GI) tract and cause tissue injury^10^. The current diagnosis of GvHD is mostly based on clinical signs in one of these three major target organs, with skin rash and diarrhoea being the most common symptoms of GvHD^11,12^.

Advances in omics fields have also warranted the identification of many blood biomarkers for post-HSCT complications over the years^13^. Existing proteomics studies mostly focus on the molecular diagnosis of GvHD post-HSCT. Both gel-based and MS-based proteomics is used, although the latter is more widely applied^14,15^. Commonly detected biomarkers primarily include acute phase proteins (APPs), whose concentrations alter in response to an inflammation^16^. These proteins could reflect organ site inflammation in acute and chronic illnesses^17^. Changes in expression of inflammatory cytokines such as IL-6, IL-8, TNF-α and IFN-γ have been described to be a promising tool for GvHD diagnosis, some of which are already routinely being used in a clinical setting^18–20^. Moreover, many past efforts involved the use of single-protein biomarkers, of which mostly reflect T cell immunological responses and the inflammatory cytokine environment in GvHD^13^.

This study presents an analysis of blood serum proteins which were significantly altered in response to HSCT, through high-performance two-dimensional gel electrophoresis (2-DE). This high-throughput technique was demonstrated to be superior to conventional methods^21^, yielding high-resolution proteomic profiles. This allows reliable comparison between states of conditions post-transplantation across patients. Longitudinal 2-DE does not require a predetermined set of proteins to be studied and can track changes in protein abundances and post-translational modifications over time. This enables unknown biomarkers to be revealed, which would permit early and accurate diagnosis of HSCT-related complications, including aGvHD. In a similar study by Ryu et al.^22^, four time points from pre-transplant to 21 days post-transplant were investigated. The expression of two positive APPs, namely, haptoglobin and C-reactive protein (CRP) were significantly up-regulated, indicating its predictive value for major transplant-related complications (MTCs)^22^. Since patients are generally more susceptible to infections during the first weeks after transplantation, the detection of proteins related to inflammation is anticipated. Examining serum proteome changes over an extended period of time (1 week pre-transplantation to 8 weeks post-transplantation), as illustrated in the present study would allow the detection of biomarkers potentially related to the onset of complications post transplantation, including GvHD, which could occur pre- or post-engraftment^23^.

## Experimental procedures

### Patient information

From August 2016 to June 2019, serum samples were collected from five acute leukaemia patients (n = 5) who were clinically diagnosed with graft-versus-host disease (GvHD) post-HSCT. Samples were collected prior to HSCT (week 0) and weekly after HSCT for 8 weeks (week 1–8) at the Institute of Medical Science, University of Tokyo. Serum samples were isolated and stored at − 80 °C within 3 h after clinical test was performed. Transportation of samples between facilities was done within 30 min on dry ice box and stored at − 80 °C until 2-DE analysis. This study was approved by the ethical committee of Tokyo Institute of Technology under the approval number of 2018071 and written informed consent from all subjects were also acquired. All research was performed in accordance with relevant guidelines/regulations. All patients were given high-dose chemotherapy combined with total body irradiation (TBI) for preparative and conditioning before stem cell transplantation. After transplantation, four out of five patients were clinically diagnosed with skin graft-versus-host disease (GvHD) while one was diagnosed with both skin and gastrointestinal (GI) tract GvHD. Table 1 shows the patient information and Table S1 shows the actual days of serum collection. Principal component analyses (PCA) were also performed to check if the variety of gender and age of patients included in this study confound the analysis (Fig. S1). Neither display a clear outlyingness of the patient of the underrepresented gender, nor is a gradient of ages visible.

**Table 1:** Information of patients included in this study.

Patient no	Age	Sex	Types of transplantation
1	45	M	CBT
2	41	M	BMT
3	50	M	CBT
4	28	M	BMT
5	28	F	CBT

Four male patients (M) and one female patients (F) were recruited in this study. Among these patients, three have undergone cord blood transplantation (CBT) and two have undergone bone marrow transplantation (BMT).

### Experimental design and statistical rationale

Samples from a total of 8 weeks post-HSCT were collected to capture the serum proteome changes during pre-engraftment, engraftment and post-engraftment periods. These are thought to be critical periods for infections and development of acute GvHD. subsequent experiments were performed in random sequences to avoid picking up batch effects. Randomization was done using simple random sampling in R, version 3.6.0^24^ with the sample() base function.

### Preparation of samples for 2-DE

In order to deplete the high abundant proteins present in human serum, Aurum serum protein mini kit (Bio-Rad) was used to selectively remove both albumin and immunoglobulin G. Later, Seppro IgY14 Spin Columns (Sigma-Aldrich) was used to remove the 14 highly abundant proteins in serum. All the samples were processed according to the manufacturer’s protocol.

Processed samples were concentrated to approximately 100 µL using Amicon Ultra-4 Centrifugal Filter Unit 3 kDa cutoff (Millipore) and subjected to 2-D Clean up kit (GE Healthcare) for removal of interfering substances. Protein quantification was then performed using 2-D Quant Kit (GE Healthcare).

### Isoelectric focusing (IEF) and two-dimensional gel electrophoresis (2-DE)

Protein samples were solubilised in Destreak Rehydration Solution (GE Healthcare) and 15 µg of protein was loaded for protein separation using Immobiline DryStrip pH 3–10 (GE Healthcare). IEF was carried out using Multiphor II (Amersham Biosciences) linked to a cooling circulator (Julabo), under these conditions: (i) 0–300 V, 1 min; (ii) 300 V, 1.5 h; (iii) 300–3500 V, 1.5 h and (iv) 3500 V, 500 h (= ∞). It was allowed to run for at least 6 h until the current value became constant. Following IEF separation, the strips were equilibrated in equilibration buffer [6 M urea; 1.5 M Tris-Hydrochloric Acid (Tris–HCl), pH 8.8; 30% v/v glycerol; 2% sodium dodecyl sulfate (SDS)] with 1% w/v DTT for reduction, then with 4.5% w/v Iodoacetamide (IAA) for alkylation.

The strips were transferred for 2-DE, on top of pre-cast NuPAGE 4–12% Bis–Tris ZOOM Gel (Invitrogen). The electrophoresis was run at 200 V, 2 mA for approximately 40 min. 2-DE gels were stained using Sypro Ruby stain (Invitrogen) and visualised using Typhoon FLA 9000 Scanner (GE Healthcare). Image Master 2D Platinum 7.0 Software (GE Healthcare) was used for spot detection and acquisition of percentage of volume contribution (%vol) of protein spots.

In total, 45 images were processed from five patients, with 9 time points per patient. One 2-DE image is produced from each sample without replicates being performed because experimental procedure was randomized, and a mixed model including both fixed and random effects was later used to determine spots that changed consistently across time points in all patients.

### Statistical analysis

All statistical analysis was performed in R, version 3.6.0^24^. A natural cubic spline with a single internal knot at the median HSCT time (t = 21 days after treatment) was fit to each spot’s log-percentage volume contribution (%vol) (Eq. 1). The justification for the knot placement is that the coefficients can be roughly interpreted as pre- and post-HSCT changes in protein abundance. Natural splines are fairly robust to knot placement, so we do not expect a large change in results if the median HSCT time changes for a larger sample. For some spots, the random intercept resulted in a singularity. These were re-estimated using a fixed effects model with a set of dummy variables for patients.

1 $$\log_ {2}\left( {\% {\text{vol}}} \right)=\upbeta_{0} + \upbeta_{1} \cdot \mathrm{ns}_{\nu=2}(\text{time})_1 + \upbeta_{2} \cdot \mathrm{ns}_{\nu=2}(\text{time})_2 + \upsilon + \epsilon \\$$

$$\upsilon \sim N\left( {0,\sigma_{\upsilon }{^{2}} } \right),\;\;\varepsilon \sim N\left( {0,\sigma_{\varepsilon }{^{2} }} \right)$$

where ns_ν = 2_ is a natural cubic spline with two degrees of freedom estimated with the splines package^24^. The conditional distribution of the log-transformed %vol of spots reasonably approximates a normal distribution. This has been observed in previous 2-DE studies on humans as well^25^. The logarithm base 2 was used for ease of interpretation in terms of doubling/halving of spot %vol. A random intercept was included to allow for baseline differences between patients. The models were fit with the lme4 package for mixed models, and p-values for the spline coefficients were obtained with the lmerTest package^26,27^. The false discovery rate (FDR) was then corrected for using the Benjamini–Hochberg procedure^28^. Spots with FDR < 0.10 are considered significant. While it is certainly conceivable that different patients show different progression over time, the five patients in this study showed similar progression, and inclusion of a random slope amounted to a model with too complex a random structure for the current number of observations.

For protein network analysis, a conditional independence network was constructed using proper ridge penalties with the rags2ridges package^29–31^. A network of all spots (Fig. 4a), as well as one using only significant spots (S3 Fig) was constructed. A local false discovery rate (lFDR) was then used to threshold non-zero edges^32^, where edges with lFDR < 0.01 were selected as non-zero. Using the igraph package^33^, neighbourhood detection was then used to reveal clusters of tightly connected spots (Fig. 4).

### In-gel tryptic digestion of significant proteins

Thirty-nine spots were excised automatically by Ettan Spot Picker (GE Healthcare). Gel plugs were picked from three different 2-DE gels where the selection of 2-DE images is based on the %vol of the spots on 2-DE images. For all spots, only one gel plug from one sample is excised. The protein gels were then reduced and alkylated using 100 mM DTT and 50 mM IAA, respectively. Gels were then incubated in Trypsin (+) solution [20 ng/µL trypsin (Trypsin Gold, Mass Spectrometry Grade, Promega), 40 mM ammonium bicarbonate, 0.2 mM HCl, 5 mM calcium chloride (CaCl_2_) and 10% acetonitrile (ACN)] for 5 min at R.T. Later, gels were incubated in Trypsin (−) solution containing the same components as Trypsin (+) solution but without trypsin at 37 °C overnight.

Following collection of supernatant, gels were incubated in ultra-pure water (Wako Pure Chemical Industries) at R.T. for 10 min. The supernatant was collected again and peptide extraction was carried out using 60% ACN, 80% ACN and 100% ACN. The collected peptide solution was concentrated by a centrifugal evaporator (CVE-2000, EYELA Tokyo Scientific Instruments) to approximately 10 µL.

The concentrated peptides were desalted using C-tip (AMR) before mass spectrometry analysis. This was performed according to the manufacturer’s guideline.

### Protein identification by LC–MS/MS

The concentrated samples were reconstituted in 0.1% trifluoroacetic acid (TFA), 2% ACN in ultra-pure water. Measurements were performed using liquid chromatography-tandem mass spectrometry (LC–MS/MS) with NIMS Proteome Discoverer 2.0 (Thermo Fisher) for analysis. Briefly, samples were injected to the inlet system with C18 column of 100 µm diameter × 150 nm length (packed with 3 µm of C18 particles). Formic acid of 0.1%/H_2_O was used for mobile phase A; 100% ACN used for mobile phase B. The samples were separated and eluted with a gradient of 5–45% in mobile phase B. The column temperature was maintained at 35 °C, with the moving flow rate maintained at 500 nL/min, and measurement of sample was performed at 20 min per sample. After separation by LC, the samples were ionised using the electrospray ionisation (ESI) technique. The generated ions were then sent to the mass spectrometer (Quadrupole orbital trap mass spectrometer, Q-Exactive, Thermo Fisher) where the mass measurements were taken. MASCOT (Version 2.5.1, Matrix Science, United Kingdom) was set to search MS/MS acquired data against Swiss-Prot database (SwissProt 2019_11, 561,568 sequences; 201,997,950 residues) using the digestion enzyme trypsin*.* The parameters are set as follows: mass range of the precursor ion was 300–5000 m/z; fragment tolerance was set to 0.02 Da and a precursor mass tolerance of 10.0 PPM; two missed cleavage sites were allowed; carbamidomethylation of Cys residues was considered as fixed modification while Met oxidation was listed as variable modifications. Identified proteins were filtered to achieve a false discovery rate (FDR) of 0.01. The data were then further filtered to exclude keratin proteins as potential contaminants from experiments, and only proteins defined as master proteins are considered. The mass spectrometry proteomics data have been deposited to the ProteomeXchange Consortium via the PRIDE partner repository with the dataset identifier PXD031349. The identified proteins were also classified using PANTHER (Version 16.0) to classify proteins according to their biological processes.

### Validation of potential biomarkers

Western Blotting was performed to validate some of the potential biomarkers identified in this study. Samples from five time points were selected: Week 0 (prior to HSCT), Week 2, Week 4, Week 6 and Week 8 (post-HSCT). Serum proteins of 10 µg was first separated by 8–12% SDS-PAGE and Precision Plus Protein WesternC Standard (Bio-Rad) was used as protein standard. Proteins were then transferred to a PVDF membrane at 15 V, 0.12 A for an hour at room temperature.

Primary antibodies used include anti-haptoglobin (ab95846, Abcam), anti-plasminogen (PA5-34677, Invitrogen), anti-retinol binding protein 4 (RBP4, PA5-80916, Invitrogen) and anti-ferritin light chain antibodies (FTL, 10727-1-AP, Proteintech) to probe for haptoglobin, plasminogen, RBP4 and ferritin light chain proteins in the samples, respectively. Primary antibodies staining was performed at 4 °C overnight. Secondary antibody staining was performed using HRP Goat Anti-Rabbit Goat IgG (AB_2795955, Southern Biotech) for an hour at room temperature. Detection of proteins was done using Merck Immobilon Western Chemiluminescent HRP substrate. Quantification of expression levels of protein bands were performed using ImageJ if the differences in intensities of bands are minimal by observation.

## Results

### 2-DE gels pre-HSCT and weekly post-HSCT

**Figure 1:**
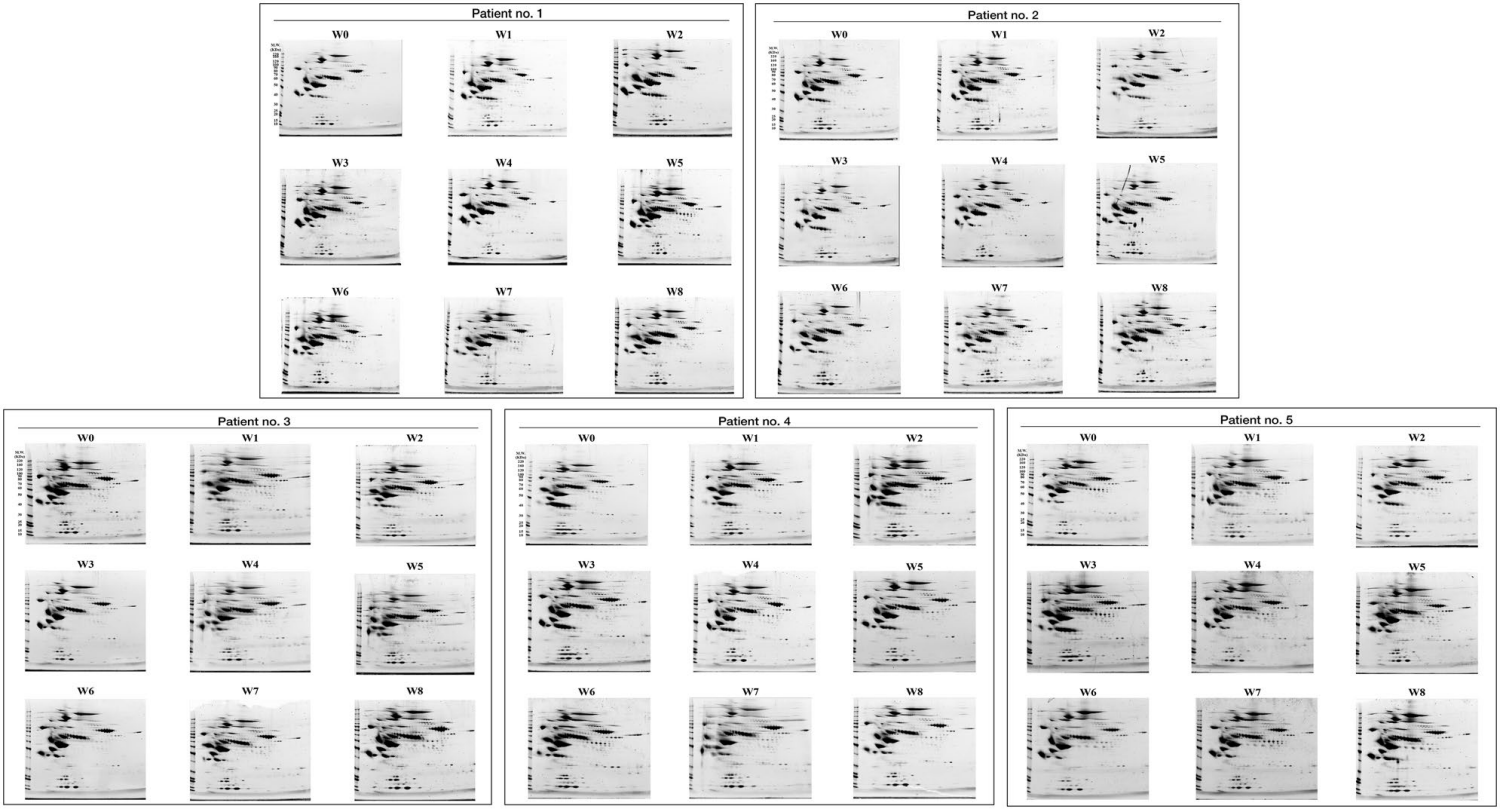
Two-dimensional gel electrophoresis (2-DE) images of serum obtained from five patients. W0 represents Week 0, sample collected before hematopoietic stem cell transplantation (HSCT); W1-W8 represent Week 1 to Week 8, samples collected weekly after patient has undergone HSCT treatment. Protein separation by 2-DE is performed using a non-linear wide range (pH 3–10) immobilized pH gradient in the first dimension and 4–12% polyacrylamide gel using 15 µg of serum protein. Data acquisition is done in randomized order.

2-DE gels of serum of five patients obtained prior to HSCT treatment, and weekly after HSCT for 8 weeks were shown in Fig. 1. Patterns of 2-DE images from five patients at different time points appear to be similar, with major proteins at mostly above 50 kDa in the acidic to neutral regions, defined by intense spot signals dominating the electrophoretic maps. Spots detection and matching of all gels were performed in Image Master 2D Platinum software resulting in 333 spot matches. The %vol of spots are then log-transformed to treat all small and big spots equally.

### Protein spots which changed over time

**Figure 2:**
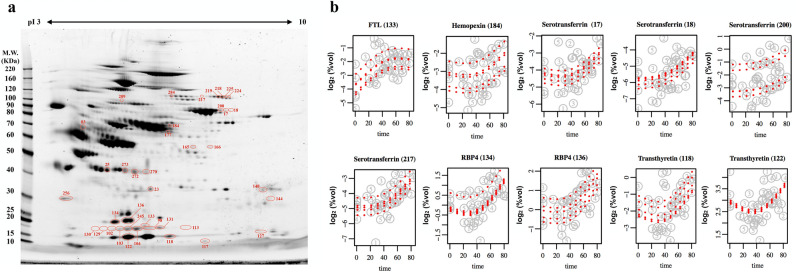
Proteins that changed significantly over time during HSCT. (**a**) Representative two-dimensional gel electrophoresis (2-DE) image showing positions of 39 significant spots changed over time during HSCT. These spots have FDR < 0.10 determined by the natural cubic spline model. The labels of spots reflect the match ID generated in Image Master 2D Platinum software. (**b**) Plots of significant spots. Plots are showing changes in spot %vol on 2-DE image over time in five patients. Names of proteins identified were labeled with spot numbers shown in brackets. FTL: ferritin light chain; RBP4: retinol-binding protein 4. The x-axis shows time in days, zero being the day of first serum collection, that is, before transplantation. The next time point was approximately 1 week after transplantation, and continued until the eighth week. Details on serum collection time is available in the supplementary data. The rest of the plots of significant spots can also be found in the supplementary data.

**Figure 3:**
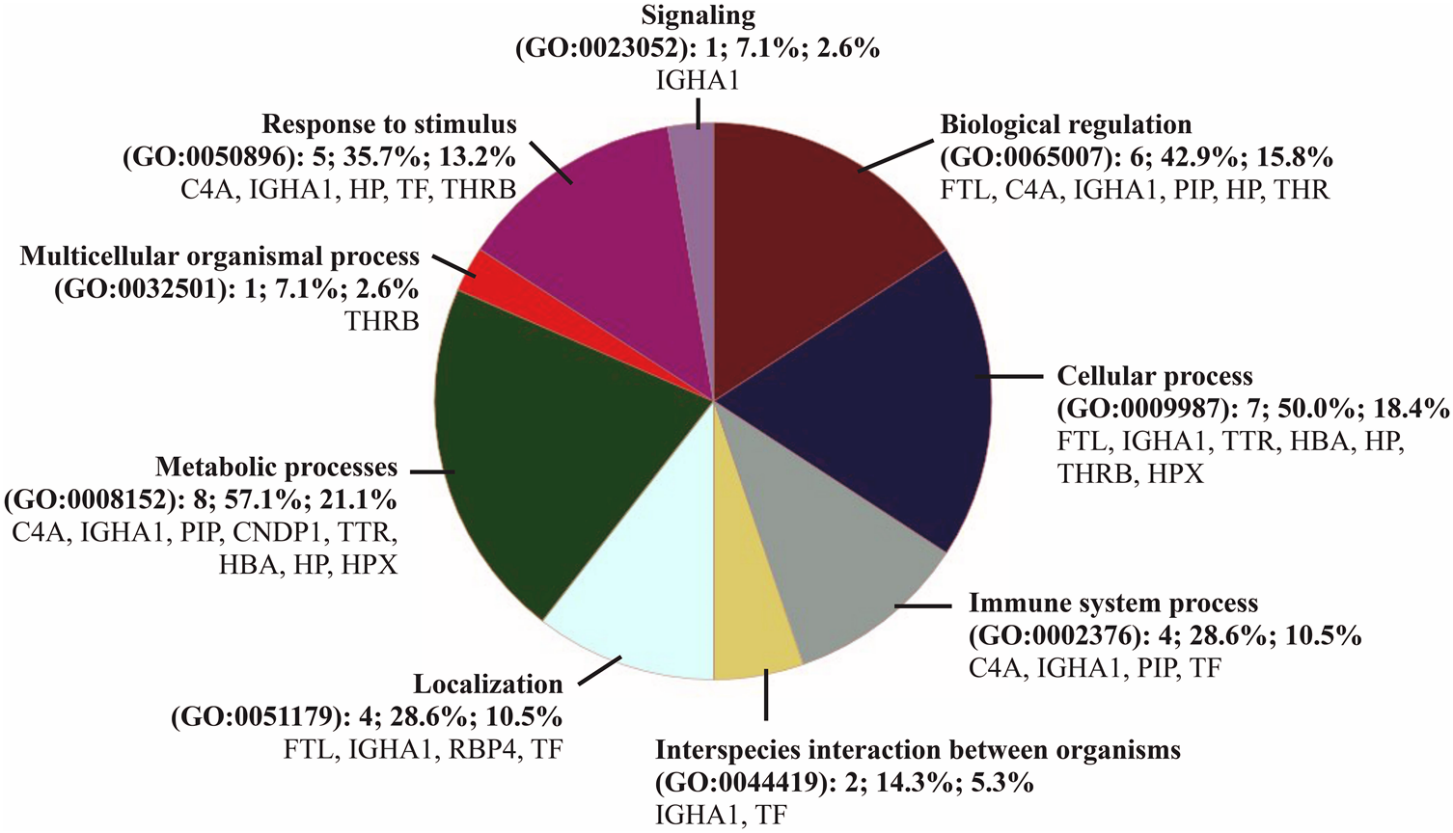
Pie chart representation of Gene Ontology (GO) for identified proteins and summarized according to biological process. Classification is done using proteins identified in Table S2. Chart includes Category name (Accession): # genes; Percent of gene hit against total # genes; Percent of gene hit against total # Process hits; Gene list for each category. FTL: ferritin light chain; HP: haptoglobin; HBA: hemoglobin subunit alpha; RBP4: retinol-binding protein 4; PLG: plasminogen; IGHA1: immunoglobulin heavy constant alpha 1; TTR: transthyretin; PIP: prolactin-inducible protein; CNDP1: beta-ala-his dipeptidase; APOH: beta-2-glycoprotein 1; TF: serotransferrin; C4A: complement C4A; THRB: prothrombin; HPX: hemopexin; C7: complement C7.

Spots which changed significantly either before or after the internal knot of the natural cubic spline model (median time until HSCT, 21 days), with a FDR value of < 0.1 were determined. A total of 39 spots were significantly altered (Fig. 2a) and these spots were subjected to mass spectrometry for protein identification (Table S2). Identified proteins were also classified using PANTHER classification system according to their biological process (Fig. 3). Most of the proteins were seen to be involved in metabolic processes (21.1%) and cellular processes (18.4%).

**Figure 4:**
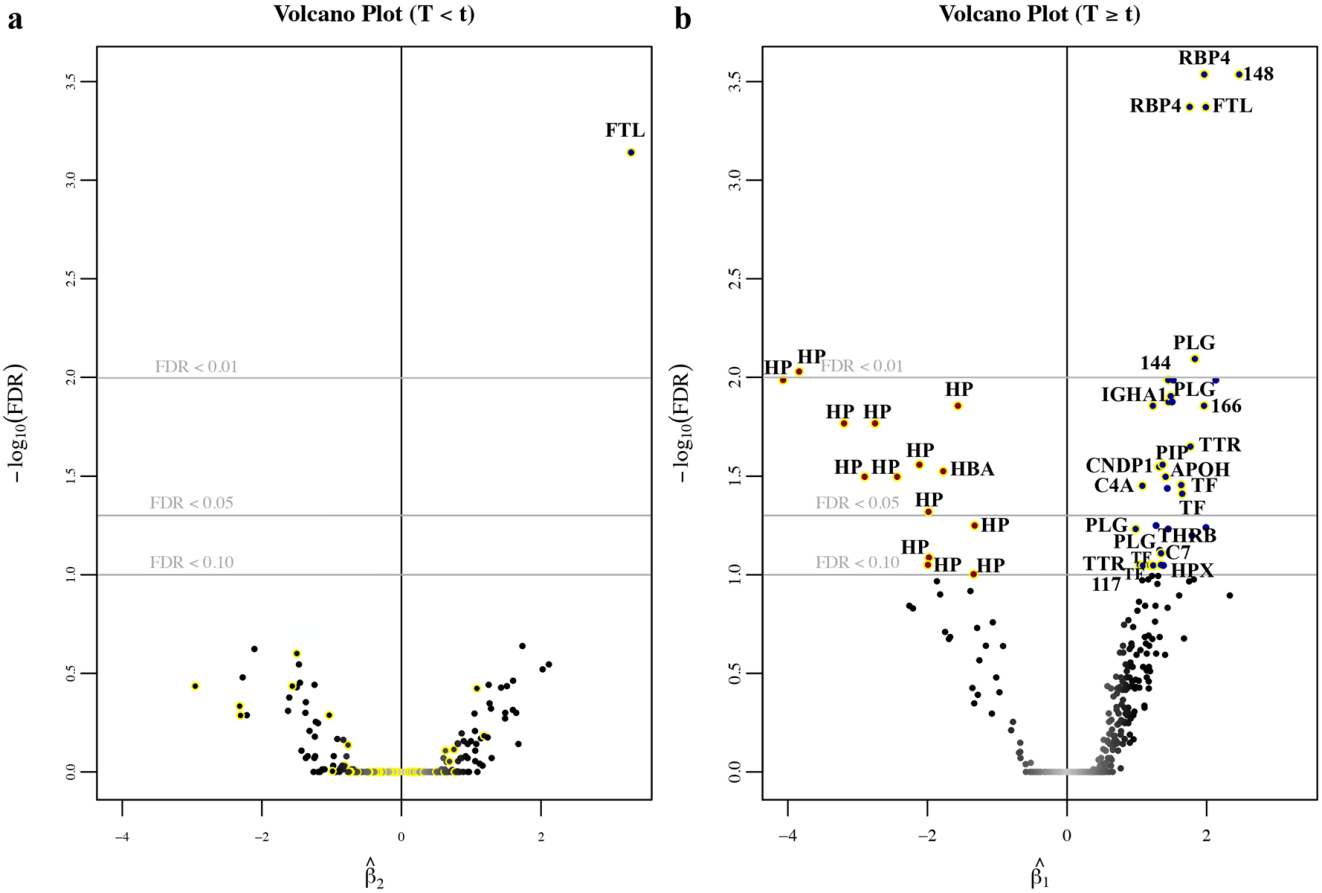
Volcano plot of the spline coefficients used to determine significant spots. The p-values for the spline coefficients were obtained in the natural spline model, using a single internal knot at the median HSCT time (t = 21 days after treatment). The false discovery rate (FDR) was then corrected for using the Benjamini–Hochberg procedure. (**a**) Volcano plot of the spline coefficient before (T < t) the internal knot, represented by β2, modelling for early changes during HSCT. (**b**) Volcano plot of the spline coefficient after (T ≥ t) the internal knot, represented by β1, modelling for later changes during HSCT. The x-axis represents the magnitude of change with the left side showing spots whose abundances are down-regulated and right side showing spots whose abundances are upregulated; y-axes represent log-transformed FDR values. The smaller the FDR value and thus higher –log10(FDR) value, the more significant the spot is.

A volcano plot showing the spline coefficient before and after internal knot is shown in Fig. 4, with plots of significant spots displayed in Figs. 2b and S2. Ferritin light chain (FTL) was significantly increased during the early stage of HSCT, as shown in Figs. 2b and 4a. In the later stage of HSCT (Fig. 4b), majority of the protein spots with decreasing abundances are identified as haptoglobin (Hp), while the most significantly upregulated proteins were identified as retinol-binding protein 4 (RBP4) and FTL.

### Protein network analysis

**Figure 5:**
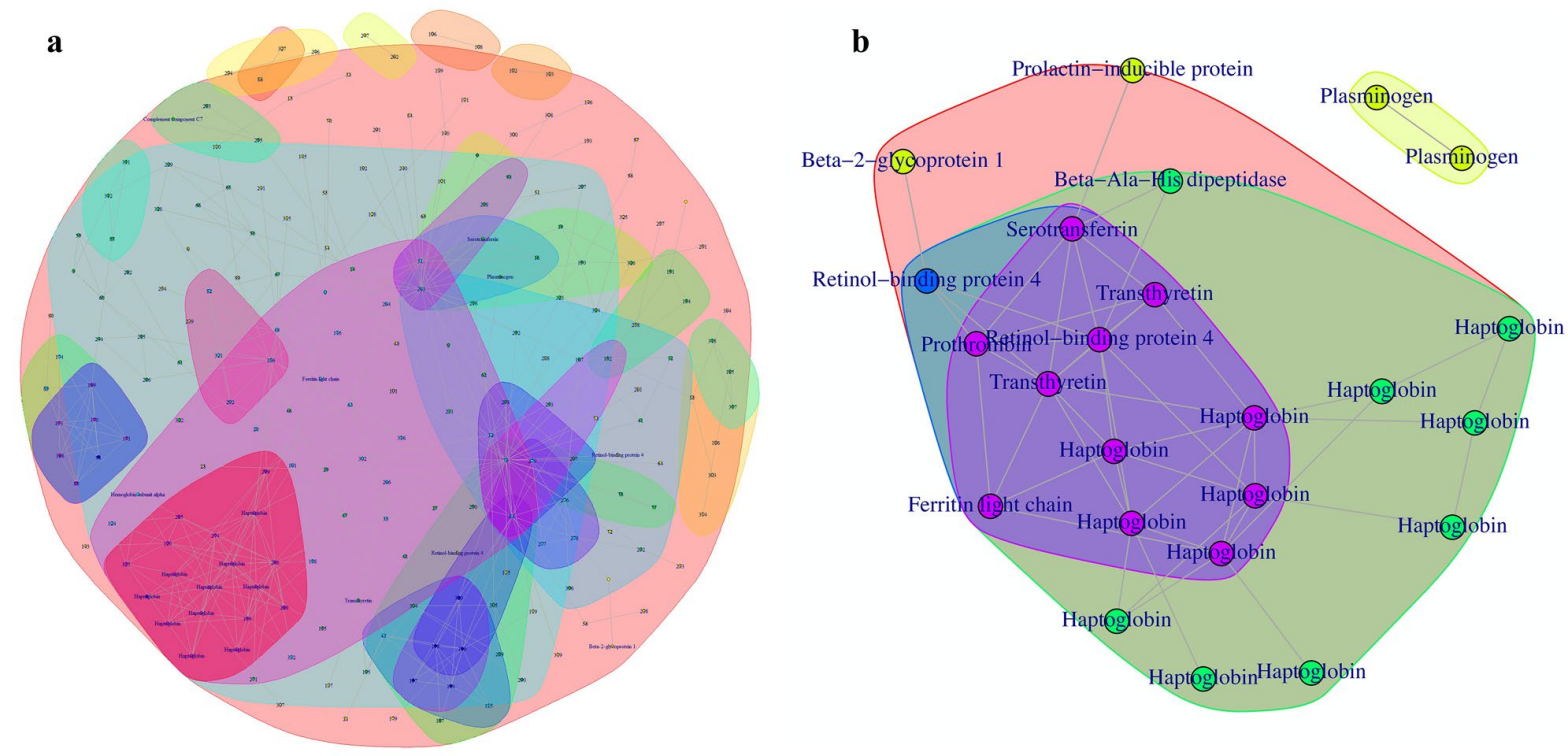
Protein network analysis. (**a**) A conditional independence network of all spots with lFDR < 0.99. (**b**) A conditional independence network among significant spots. Partial correlations lower than 20% were removed. Each community is represented by different colors, enclosing proteins that are closest to one another in the network.

A conditional independence network was used to look at partial correlations among all spots (with lFDR < 0.99) and among the significant spots, as shown in Fig. 5a,b, respectively. The community detection analysis revealed clusters of tightly connected spots, indicating both positive and negative direct relationships between protein spots. The direction of the relationship remains unknown. Figure 5a revealed a community of spots, many of which were identified as fragments of haptoglobin (shown by cluster colored in dark pink). This suggests that the remaining unidentified spots in the community are either also parts of haptoglobin, or are proteins strongly associated with haptoglobin and may have similar physiological functions during HSCT. When considering partial correlations between proteins that changed significantly over time (FDR < 0.10) (Fig. 5b), a graph of partial correlations greater than 0.20 revealed that most of the clusters overlapped, with the center cluster (colored in purple) having proteins densely connected to each other. These proteins have strong partial correlations to each other and are thought to be significantly affected during HSCT. Moreover, plasminogen is clustered separately from the other communities, representing plasminogen are conditionally independent of the %vol of other spots. This suggests that the changes observed over time are also independent.

### Validation of potential biomarkers

**Figure 6:**
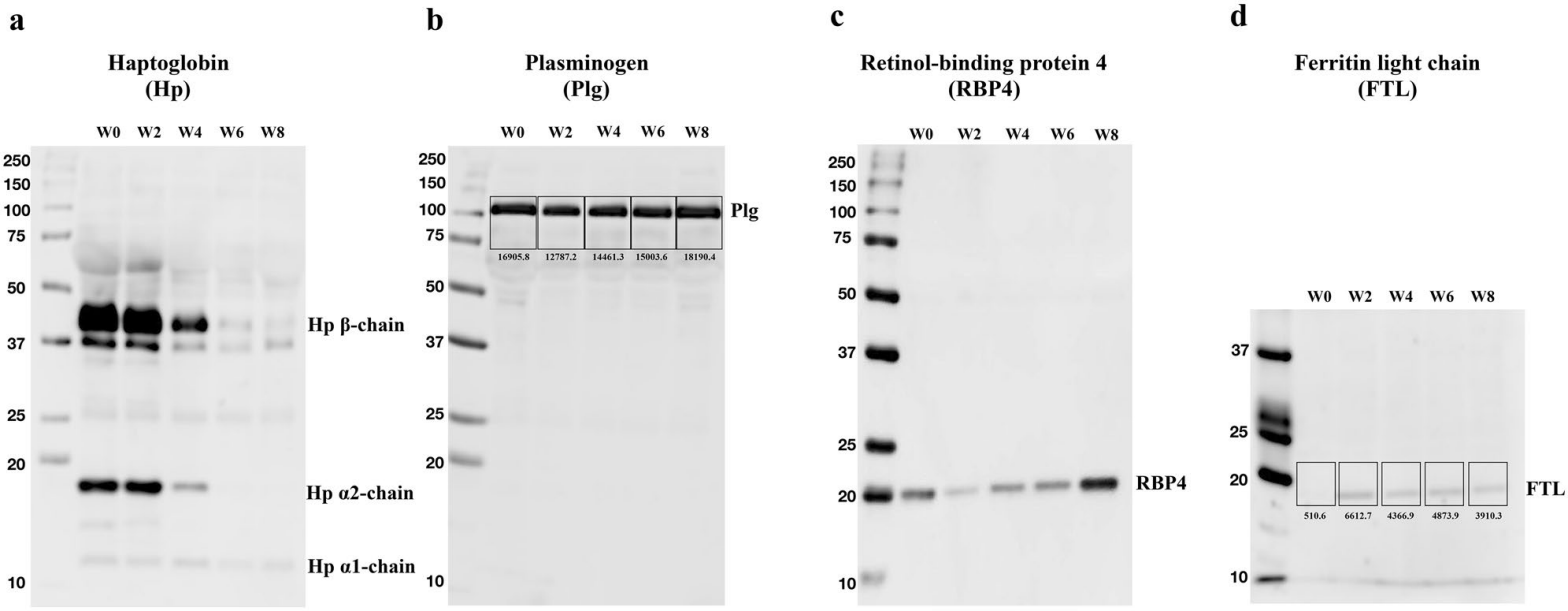
Post-transplant serial changes in four serum proteins from Patient no.1. Serum samples collected over time from patient no.1 were subjected to western blotting analysis. Serum proteins of 10 µg was first separated by 8–12% SDS-PAGE and Precision Plus Protein WesternC Standard (Bio-Rad) was used as protein standard. Proteins were then transferred to a PVDF membrane and stained with antibodies. Primary antibodies were diluted at 2000-fold except for FTL (1000-fold). HRP Goat Anti-Rabbit IgG was used as secondary antibody in 2000-fold dilution except for RBP4 (10,000-fold). Signals of bands were quantified using ImageJ software.

The results of Western Blotting analysis corroborate with the results of 2-DE. The expression of haptoglobin before HSCT and 2 weeks post-HSCT remained similar, but notably diminished from Week 4 to Week 8 (Fig. 6a). The signal of plasminogen diminished 2 weeks post-HSCT before it increases until Week 8 (Fig. 6b). As for the expression of RBP4, there was a notable decrease in its signal 2 weeks post-HSCT before it increases at Week 8 (Fig. 6c). It is noteworthy that the expression of RBP4 at Week 8 was stronger than its expression before transplantation. Finally, validation of increased expression of ferritin light chain (FTL) was also performed (Fig. 6d). The expression of FTL was low prior to HSCT, but increases 2 weeks post-HSCT. The expression remains higher than pre-HSCT even after 8 weeks post-HSCT.

## Discussion

The increased plasminogen found in our study may indicate hematopoietic regeneration and/or stem cell mobilisation^34,35^. Plasmin, a serine protease, is the primary enzyme for fibrinolysis, while plasminogen itself is an inactive precursor of plasmin^36^. Previous reports have shown that the plasminogen fibrinolytic pathway is essential for hematopoietic regeneration, by ensuring sufficient supply of mature and immature hematopoietic cells in the circulation. Deletion of the Plg gene in mice has prevented hematopoietic stem cells from entering cell cycle, eventually leading to death of the mice^34^. Therefore, we hypothesize that the elevated expression of plasminogen a month post-transplantation in our study could indicate hematopoietic regeneration after engraftment. In the result of Western Blotting analysis (Fig. 6b), plasminogen level diminished 2 weeks post-HSCT, and increased later during hematopoietic regeneration. Furthermore, the community detection analysis of only significant spots (Fig. 5b) also revealed that the changes in expression of plasminogen was independent of the trends of other significant spots, further confirming our postulation that plasminogen was an indicator of successful engraftment after HSCT in patients.

Iron overload is commonly observed in HSCT recipients, particularly in acute leukaemia patients^37^ This is caused by regular red blood cells transfusion pre-transplantation, followed by continued transfusion therapy post-transplantation^38,39^. This was also reflected in the present study, where the expression of ferritin was elevated during early post-transplantation. After transplantation, the expression of ferritin light chain (FTL) remains higher than before HSCT (Fig. 6d). Ferritin is the major protein for iron storage and often used as an indicator of cellular iron stores^40^. Since iron overload causes increased risk of infection and other post-transplantation complications such as vena-occlusive disease (VOD), graft-versus-host disease (GvHD), hepatic dysfunction and metabolic and insulin resistance syndrome^41,42^, several past studies have also demonstrated the association between elevated ferritin levels and poor outcomes post-HSCT^43–46^. Other evidence of iron overload in the subjects of our study was the elevated expression of serotransferrin. Serotransferrin (i.e., transferrin), binds to iron with high affinity and transports iron to the liver, spleen and bone marrow^47^. Typically, its level rises with iron deficiency, and drops with iron stores^48^. Interestingly, in the present study, there was a rise in both the expression of ferritin and serotransferrin. Such condition has been associated with metabolic disorders and insulin resistance^49^.

Hemopexin, a plasma protein which has the highest binding affinity for heme^50^ was elevated in this study. In a situation where there is free heme in circulation, the free heme would catalyze free radical reactions, therefore promoting oxidative damage, and the body would then defend itself by inducing hemopexin, the plasma scavengers of heme^51,52^. Therefore, the increased expression of hemopexin found in this study may have a protective role against heme toxicity. In an animal study using mice model of heme overload, it was shown that hemopexin is essential in preventing vascular inflammation and vaso-occlusion^53^. In short, the high levels of hemopexin could indicate high levels of free heme in the circulation as a result of iron overload, and high levels of free heme have been associated with endothelial cell injury, vascular inflammatory disorder and hemolytic disorder^54,55^.

We also observed a down-regulation of hemoglobin subunit alpha (HbA1), a small molecular weight protein involved in oxygen transport from the lungs to other tissues^56^. Low HbA1 suggests increased risk of hypoglycemia, possibly associated with liver disease and increased insulin resistance^57–59^. This could be a result of a combination of abnormal red blood cell turnover and function, such as anemia and iron overload, which are both fairly common complications after stem cell transplantation^58,60^.

The majority of proteins decreasing in abundance were identified as haptoglobin. This decrease was also validated by Western Blotting analysis (Fig. 6a). Post-translational modifications of haptoglobin were also seen as multiple proteins at different positions of 2-DE were identified as haptoglobin. These proteins displayed a shift in pI values (Fig. 2a) and exhibited consistent change in expression. In healthy individuals, haptoglobin produced in the liver binds to free hemoglobin to prevent free hemoglobin-induced vascular injury^61^. While increased expression of haptoglobin was seen in patients with chronic graft-versus-host disease (GvHD)^62^, haptoglobin levels falling below lower limit of normal range is often associated with post-transplant thrombotic microangiopathy (TA-TMA) and autoimmune hemolytic anemia (AIHA)^63–65^.

Other than serotransferrin, our study has also identified another two negative acute phase proteins (APPs), known as retinol-binding protein 4 (RBP4) and transthyretin, where the synthesis of these proteins typically decrease in response to an inflammatory reaction^66^. Among the proteins identified in this study, RBP4 was the most significantly up-regulated protein early post-HSCT. The up regulated expression was also apparent in Week 9 post-HSCT in the result of Western Blotting analysis (Fig. 6c). Transthyretin transports thyroxine and RBP4 in the serum^67^, while RBP4 produced in the liver, is a major transporter for retinol (Vitamin A alcohol) to the peripheral tissues^68^. RBP4 was mainly synthesized in the liver, but not exclusively, and has been found to be expressed in adipocytes^69^. High expression of RBP4 has been associated with low necroinflammatory activity, low NAFLD activity and low fibrosis score^70^. While decreased levels of RBP4 should be alarming, its continually elevated expression as shown in the present study may be a sign of insulin resistance. RBP4-over-expressing mice displayed insulin-resistant, glucose-intolerant, increased adipose tissue macrophages and CD4 T-cell infiltration^71^. To the best of our knowledge, insulin resistance was rarely reported to be an immediate effect following transplantation.

In addition, it is thought that systemic insulin resistance after transplantation may be due to adipose tissue inflammation triggered by RBP4 in vivo^71^. In a case study of chronic GvHD adult patients, partial lipodystrophy was reported, where residual damage to muscle and fat tissue was detected^72^. Moreover, distinct patterns of fat distribution in patients were also reported and have been associated with chronic sclerodermatous GvHD^73^, which may explain the involvement of the skin in the development of aGvHD in the patients included in our studies. Furthermore, it has been suggested that total body irradiation and/or intensive chemotherapy prior to HSCT may contribute to the damage of adipocytes in subcutaneous fat, limiting the body’s lipid-storage capacity^74,75^. These studies have found association between insulin resistance, dyslipidemia and partial lipodystrophy with cGvHD. Even though such metabolic disorders after HSCT are typically considered as long-term consequences^76,77^, we herein describe that insulin resistance may occur during the early period after HSCT and may be associated with subsequent aGvHD development. This is the first study to report RBP4 and its elevation after HSCT as a marker of insulin resistance which may be the predominant manifestation of GvHD.

Furthermore, an enzyme β-ala-his dipeptidase (CNDP1), or better known as serum carnosinase, also showed changes in expression in serum proteome of patients after transplantation. Many previous researches have focused on the association of this gene with human diabetic nephropathy^78–80^. It was demonstrated that low carnosinase activity has a protective effect against adverse effects of high glucose levels on kidney, resulted from an increase of carnosine in blood^81^. Although it is not understood why serum carnosinase showed increased in expression in our study, it increases the degradation of carnosine, which may have result in decreased renoprotective properties.

While the overall survival of HSCT recipients have improved over the years, various post-transplant complications including graft-versus-host disease (GvHD) remain problematic as the quality of life of HSCT survivors are severely affected. One of the major advantages of the present study was its ability to monitor the patient’s conditions over the span of 9 weeks (8 weeks post-HSCT), allowing a more thorough analysis of the changes in serum proteome after HSCT, including GvHD manifestation both pre- and post- engraftment. The current study, however, is restricted by its limited observations, hindering the implementation of clustering strategy (e.g. classifying individuals with good and adverse outcome) for a more comprehensive analysis. Moreover, the current findings are also limited by the potential influence of therapeutic modalities before HSCT. An approach to examine serum proteome changes due to only high dose of chemotherapy and radiotherapy and include non-GvHD patients in the study could be considered in future work to understand if the current findings are only observed in HSCT recipients who developed GvHD.

Although the present study is underpowered at only 5 patients, a number of well-established biomarkers, along with several new ones were identified. In addition, the protein network analysis revealed cluster of proteins and possible relationships with other non-significant spots (Fig. 5a). This enables the demonstration of relationships with other proteins which potentially have similar physiological function after HSCT. Several significant proteins which were unidentified and identified with low confidence due to low protein amount in the digested gel plugs could be validated further in future work.

During the process of rebuilding the immune system with donor cells, our observations showed that the patients included in the present study may develop hyperferritinemia, hemolytic anemia, thrombosis and insulin resistance despite successful engraftment. Although insulin resistance was rarely associated with acute graft-versus-host disease, based on our analysis, it is possible that RBP4 may have attracted macrophages to the adipocytes causing adipose tissue inflammation, thus damage to muscle and fat tissue. Changes in tissue is also thought to have linkage with the clinical manifestation of cutaneous GvHD. After which, the body’s lipid storage capacity becomes limited following tissue injury, eventually leading to insulin resistance. Nonetheless, it is also possible that it was the result of the interfered adipocyte insulin signalling after the local inflammation of adipocytes^82^. In addition, it is also important to note that insulin resistance marked by the increased levels of RBP4 may also be a result of iron overload, hemolytic anemia and thrombosis, as these were all suggested in our study. Furthermore, our protein network analysis demonstrated the direct relationships between these proteins (Fig. 5b). Even though it is not completely understood as to how insulin resistance is associated with GvHD, we postulate that the pathogenesis of insulin resistance may have prompted tissue damage in that process. As there are various manifestations of aGvHD, our study now present insulin resistance as one of the manifestations of GvHD.

In conclusion, during the process of rebuilding the immune system with donor cells, HSCT recipients may develop hyperferritinemia, hemolytic anemia and insulin resistance despite successful engraftment. RBP4 is a reliable marker of insulin resistance, and is possibly associated with the onset mechanism of acute graft-versus-host disease (aGvHD) and/or skin GvHD. Although the cause of insulin resistance is not fully understood, it is thought to be associated with adipocytes inflammation induced by RBP4, iron overload and hemolytic anemia after hematopoietic stem cell transplantation. These manifestations are often only considered in the context of late complications, such as in chronic GvHD. The present study, however, has demonstrated that insulin resistance and metabolic complications could be immediate complications after transplantation and are associated with aGvHD.

## Supplementary Information

Supplementary Information.

## Data Availability

All data are available within the manuscript and supplemental data.

## Abbreviations

HSCT: Hematopoietic stem cell transplantation
GvHD: Graft-versus-host disease
aGvHD: Acute graft-versus-host disease
2-DE: Two-dimensional gel electrophoresis
RBP4: Retinol-binding protein 4
APP: Acute phase proteins
IL: Interleukin
TNF: Tumor necrosis factor
CRP: C-reactive protein
MTCs: Major transplant-related complications
TBI: Total body irradiation
GI: Gastrointestinal
CBT: Cord blood transplantation
BMT: Bone marrow transplantation
FDR: False discovery rate
lFDR: Local false discovery rate
FTL: Ferritin light chain
HRP: Horseradish peroxidase
Hp: Haptoglobin
Plg: Plasminogen
VOD: Vena-occlusive disease
HbA: Haemoglobin subunit alpha
TA-TMA: Post-transplant thrombotic microangiopathy
AIHA: Autoimmune hemolytic anemia
β2GP1: Beta-2-glycoprotein 1
APS: Antiphospholipid syndrome
SLE: Systemic lupus erythematous
NAFLD: Non-alcoholic fatty liver disease
CNDP1: β-Ala-his dipeptidase
