# Supplementary material for: Longitudinal proteomics study of serum changes after allogeneic HSCT reveals potential markers of metabolic complications related to aGvHD
Source: Sci Rep. 2022 Aug 17;12:14002. doi: 10.1038/s41598-022-18221-9 (PMC9385631; doi:10.1038/s41598-022-18221-9)
Supplement: Supplementary file 1 — Supplementary Information. [file 41598_2022_18221_MOESM1_ESM.pdf]

Supplemental Information: Tables and Figures

Title

Longitudinal proteomics study of serum changes after allogeneic HSCT reveals potential markers of metabolic complications related to aGvHD

Authors

Sing Ying Wong, Seiko Kato, Frans Rodenburg, Arinobu Tojo, Nobuhiro Hayashi

Table S1. Days of serum collection. Day of patient receiving HSCT is denoted as Day 0. Hence, Day -14 would indicate 14 days before HSCT; Day 7 would indicate 7 days after HSCT.

Patient	Day
1	-14
	7
	14
	22
	28
	35
	42
	49
	56
2	-24
	7
	14
	21
	27
	35
	42
	49
	56
3	-8
	7
	14
	21
	28
	35
	42
	49
	56
4	-21
	7
	14
	21
	28

	35
	42
	50
	56
5	-14
	7
	14
	21
	28
	35
	42
	49
	56

Table S2. Identified proteins which changed significantly over time.

Spot ID	Accession	Description	Coverage	calc. pI	MW [kDa]	#PSMs	#Peptides	# Unique Peptides	Mascot Score
17	P02787	Serotransferrin	6.16	7.12	77.01	4	4	4	131.65
18	P02787	Serotransferrin	1.72	7.12	77.01	1	1	1	36.4
23	P12273	Prolactin-inducible protein	8.22	8.05	16.56	1	1	1	28.4
83	Q96KN2	Beta-Ala-His dipeptidase	20.32	5.3	56.67	12	9	9	304
	P01011	Alpha-1-antichymotrypsin	16.78	5.52	47.62	8	6	6	209.6
	P01042	Kininogen-1	7.30	6.81	71.91	7	5	5	198.8
	P01019	Angiotensinogen	8.66	6.32	53.12	4	3	3	190.1
	P04180	Phosphatidylcholine-sterol acyltransferase	5.68	6.11	49.55	2	2	2	83.2
	P10909	Clusterin	2.45	6.27	52.46	1	1	1	63
	Q9Y6R7	IgGFc-binding protein	0.56	5.34	571.64	1	1	1	50.3
	P05543	Thyroxine-binding globulin	2.17	6.3	46.30	1	1	1	46.7
	P12273	Prolactin-inducible protein	8.22	8.05	16.56	2	1	1	41.7
	P51884	Lumican OS=Homo sapiens	2.66	6.61	38.41	1	1	1	35.5
95	P00738	Haptoglobin	27.09	6.58	45.18	28	13	13	743.6
	P06727	Apolipoprotein A-IV	13.13	5.38	45.37	5	5	5	69.7
	P02766	Transthyretin	8.84	5.76	15.88	1	1	1	51
	Q02413	Desmoglein-1	2.86	5.03	113.68	2	2	2	18.36
98	P00738	Haptoglobin	8.62	6.58	45.18	3	3	3	65.7

	P02766	Transthyretin	8.84	5.76	15.88	1	1	1	59.5
	P12273	Prolactin-inducible protein	8.22	8.05	16.56	1	1	1	38.2
	P15924	Desmoplakin	0.70	6.81	331.57	2	2	2	36.84
102	P00738	Haptoglobin	3.20	6.58	45.18	1	1	1	43.3
103	P00738	Haptoglobin	7.39	6.58	45.18	3	2	2	75
	P12273	Prolactin-inducible protein	8.22	8.05	16.56	1	1	1	46.4
104	P00738	Haptoglobin	11.58	6.58	45.18	6	6	6	163.4
113	P00738	Haptoglobin	3.20	6.58	45.18	1	1	1	27.1
117	Unidentified								
118	P02766	Transthyretin	24.49	5.76	15.88	2	2	2	34.8
122	P02766	Transthyretin	73.47	5.76	15.88	32	12	12	1151.6
	P16402	Histone H1.3	4.07	11.02	22.34	1	1	1	16.1
127	P69905	Hemoglobin subunit alpha	14.79	8.68	15.25	2	2	2	68.1
	P02042	Hemoglobin subunit delta	8.84	8.05	16.05	1	1	1	47.8
129	P00738	Haptoglobin	3.20	6.58	45.18	1	1	1	32.4
130	P00738	Haptoglobin	6.90	6.58	45.18	4	4	4	188.3
131	P00738	Haptoglobin	7.14	6.58	45.18	4	4	4	162
133	P02792	Ferritin light chain	8.57	5.78	20.01	1	1	1	96.5
	P02790	Hemopexin	2.38	7.02	51.64	1	1	1	32.9
134	P02753	Retinol-binding protein 4	10.45	6.07	23.00	4	2	2	122.3
136	P02753	Retinol-binding protein 4	19.90	6.07	23.00	8	4	4	222.1

144	Unidentified								
148	Unidentified								
165	P02749	Beta-2-glycoprotein 1	4.06	7.97	38.27	1	1	1	54.8
	P02790	Hemopexin	4.76	7.02	51.64	2	2	2	48.2
166	Unidentified								
177	P01876	Immunoglobulin heavy constant alpha 1	14.45	6.51	37.63	7	4	4	216.5
	P02790	Hemopexin	7.14	7.02	51.64	3	3	3	116.4
	P20742	Pregnancy zone protein	0.54	6.38	163.76	1	1	1	45.4
184	P02790	Hemopexin	17.32	7.02	51.64	10	7	7	316
	P20742	Pregnancy zone protein	0.74	6.38	163.76	1	1	1	39
	P01023	Alpha-2-macroglobulin	0.75	6.46	163.19	1	1	1	
	O75339	Cartilage intermediate layer protein 1	1.27	8.41	132.48	1	1	1	17.6
200	P02787	Serotransferrin	29.08	7.12	77.01	25	18	18	762.8
209	P0C0L4	Complement C4-A	5.50	7.08	192.66	8	7	1	240.3
	P0C0L5	Complement C4-B	5.28	7.27	192.63	8	7	1	238.6
	P00450	Ceruloplasmin	4.60	5.72	122.13	4	4	4	131
	P16402	Histone H1.3	4.07	11.02	22.34	1	1	1	17.9
217	P02787	Serotransferrin	4.15	7.12	77.01	3	3	3	83.5
	P00751	Complement factor B	1.44	7.06	85.48	1	1	1	63.9
218	P00747	Plasminogen	8.40	7.24	90.51	8	7	7	190.4

	P12273	Prolactin-inducible protein	8.22	8.05	16.56	1	1	1	32.9
	P46736	Lys-63-specific deubiquitinase BRCC36	3.16	5.92	36.05	1	1	0	26.1
	O15446	DNA-directed RNA polymerase I subunit RPA34	1.18	8.51	54.95	1	1	0	24.3
	Q6ZP82	Coiled-coil domain-containing protein 141	0.41	5.58	166.16	1	1	0	24.3
	O43150	Arf-GAP with SH3 domain, ANK repeat and PH domain-containing protein 2	0.80	6.68	111.58	1	1	0	22.8
219	P00747	Plasminogen	12.10	7.24	90.51	9	9	9	242
224	P00747	Plasminogen	2.59	7.24	90.51	2	2	2	56.1
225	P00747	Plasminogen	6.30	7.24	90.51	5	5	5	107.9
	P31025	Lipocalin-1	6.25	5.58	19.24	1	1	1	25.6
	Q8NGK0	Olfactory receptor 51G2	2.55	8.59	34.99	1	1	1	23.7
	O75339	Cartilage intermediate layer protein 1	1.27	8.41	132.48	1	1	1	17.1
245	P00738	Haptoglobin	6.90	6.58	45.18	4	4	4	180.6
	P16402	Histone H1.3	4.07	11.02	22.34	1	1	1	22.7
256	P00734	Prothrombin	1.45	5.9	69.99	1	1	1	51.2
	O14654	Insulin receptor substrate 4	0.80	8.44	133.69	1	1	1	22.1
270	P00738	Haptoglobin	16.50	6.58	45.18	10	7	7	398.5
272	P00738	Haptoglobin	27.09	6.58	45.18	20	10	10	606.2
	P25311	Zinc-alpha-2-glycoprotein	3.36	6.05	34.24	1	1	1	29.7
	P12273	Prolactin-inducible protein	5.48	8.05	16.56	1	1	1	24.2

	Q9UKB3	DnaJ homolog subfamily C member 12	3.54	5.71	23.40	1	1	1	23.2
273	P00738	Haptoglobin	11.82	6.58	45.18	5	5	5	239.6
	P06727	Apolipoprotein A-IV	11.87	5.38	45.37	7	5	5	88.4
284	P10643	Complement component C7	5.10	6.48	93.46	5	3	3	137.4
	P06681	Complement C2	1.60	7.42	83.21	1	1	1	56.6

Spot ID are spot matches according to Fig. 2a; Accession describes the accession no. obtained from the Swiss-Prot database human (Homo sapiens); Description represents the protein name; Coverage is the percentage of protein sequence covered by identified peptides; Calc. pI and MW represent the theoretically calculated isoelectric point and calculated molecular weight of the proteins, respectively; PSMs represents the peptide spectrum for the protein; #Peptides displays the number of distinct peptide sequences in the protein group; #Unique peptides represent the number of peptide sequences that are unique to a protein group; Mascot score describes the sum of the ion scores of all peptides match those in the protein database of the Mascot server.

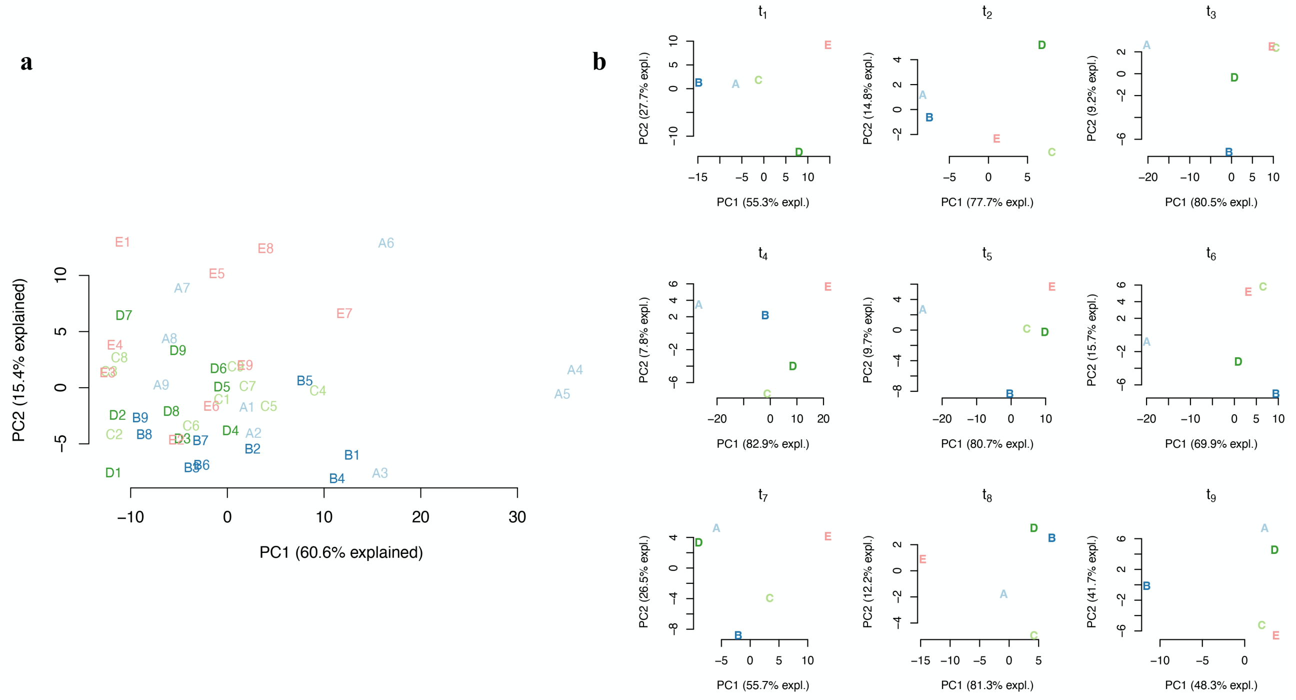

Figure S1. Principal component analyses (PCA) for potential confounding factors. a PCA of all observations. A - E represent patients while 1 - 9 represent time (week 0 to week 8). **b** PCA for each time point: t1 - t9 representing week 0 to week 8. No clear outlyingness of patient is observed in these analyses displaying the unlikelihood of gender and ages as confounding factors.

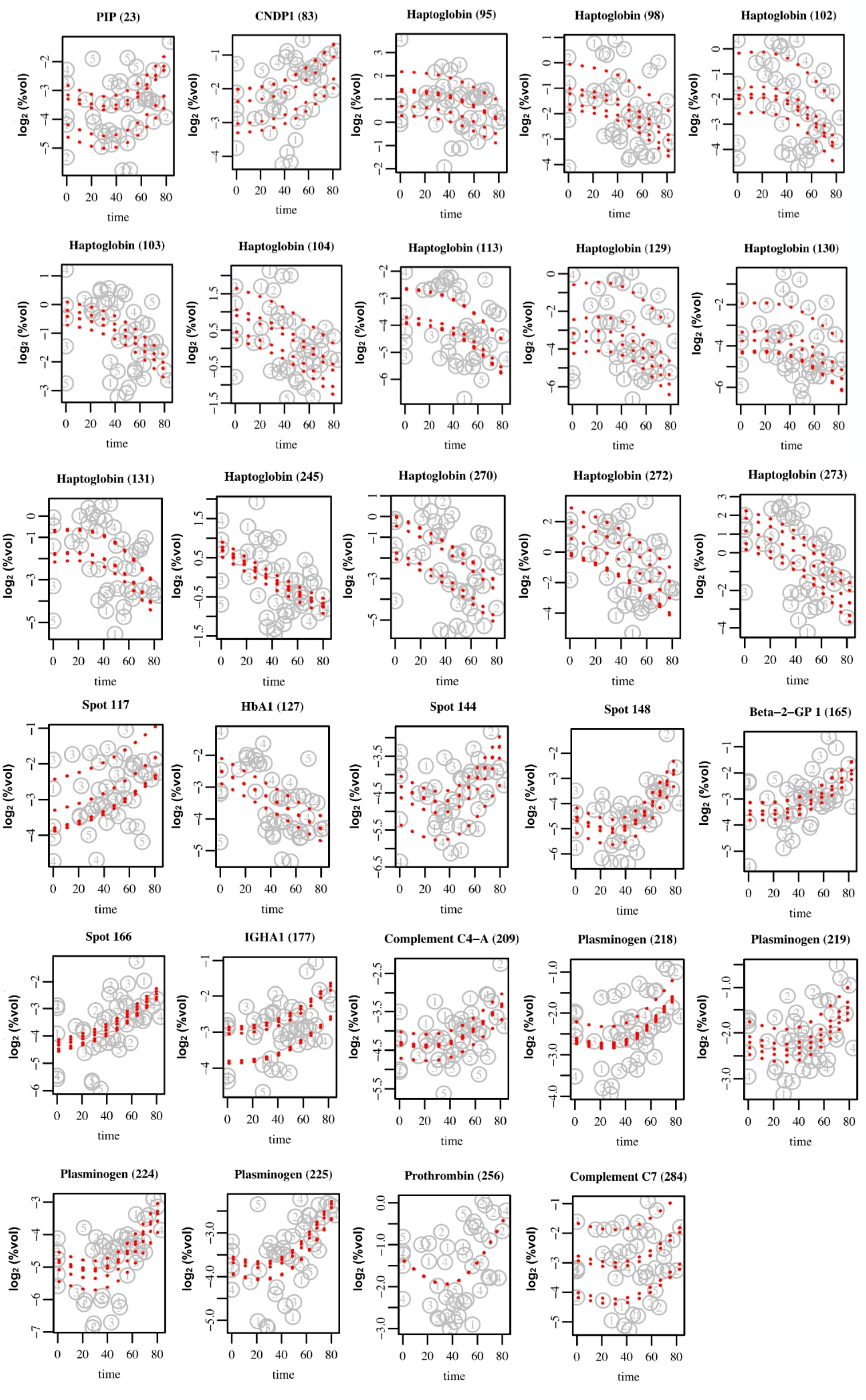

Figure S2. Plots of significant spots. Plots are showing changes in spot %vol on 2-DE image over time in five patients. The x-axis shows time in days, zero being the day of first serum collection, that is, before transplantation. The next time point was approximately one week after transplantation and continued until the eighth week. Names of proteins identified were labeled with spot numbers shown in brackets. PIP: prolactin-inducible protein; CNDP1: Beta-Ala-His dipeptidase; HbA1: hemoglobin subunit alpha; Beta-2-GP1: Beta-2-glycoprotein 1; IGHA1: immunoglobulin heavy constant alpha 1.

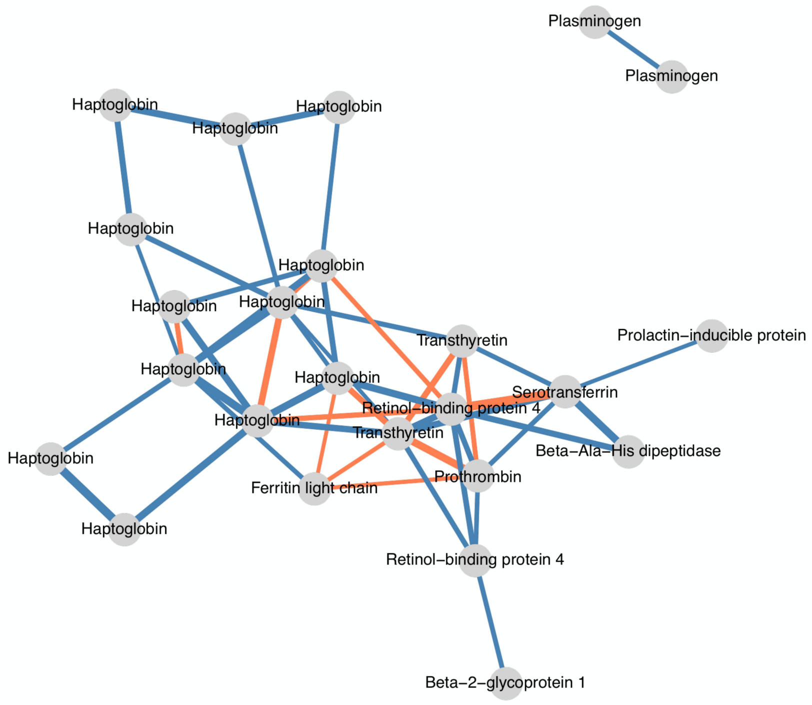

Figure S3. Conditional independence network of significant spots. This shows the top 50 partial correlations among the spots that changed significantly during HSCT.

Supplemental Information: Fragment matches and spectrum of proteins identified with less than 2 unique peptides

Title

Longitudinal proteomics study of serum changes after allogeneic HSCT reveals potential markers of metabolic complications related to aGvHD

Authors

Sing Ying Wong, Seiko Kato, Frans Rodenburg, Arinobu Tojo, Nobuhiro Hayashi

Description

Several proteins identified in this study (Table S2) display low identification confidence with low unique peptide number and mascot score. The fragment matches and spectrum of these proteins are provided as supplemental information.

Spot 18: Serotransferrin (P02787)

Fragment matches						
**#1**	**b^+**	**b^{2+}**	**Seq.**	**y^+**	**y^{2+}**	**#2**
1	148.04268	74.52498	M-Oxidation			12
2	311.10600	156.05664	Y	1347.69433	674.35080	11
3	424.19007	212.59867	L	1184.63100	592.81914	10
4	481.21153	241.10940	G	1071.54694	536.27711	9
5	644.27486	322.64107	Y	1014.52547	507.76637	8
6	773.31745	387.16237	E	851.46214	426.23471	7
7	936.38078	468.69403	Y	722.41955	361.71341	6
8	1035.44920	518.22824	V	559.35622	280.18175	5
9	1136.49687	568.75208	T	460.28781	230.64754	4
10	1207.53399	604.27063	A	359.24013	180.12370	3
11	1320.61805	660.81266	I	288.20302	144.60515	2
12			R	175.11895	88.06311	1

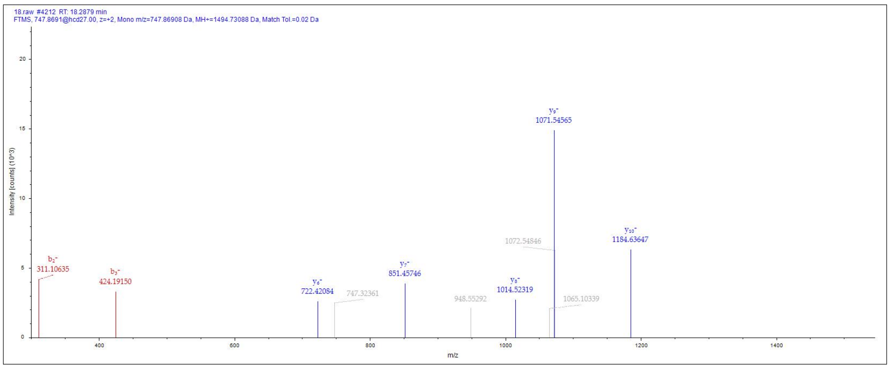

Spot 23: Prolactin-inducible protein (P12273)

Fragment matches						
**#1**	**b^+**	**b^{2+}**	**Seq.**	**y^+**	**y^{2+}**	**#2**
1	102.05496	51.53112	T			12
2	201.12337	101.06532	V	1182.72048	591.86388	11
3	329.18195	165.09461	Q	1083.65207	542.32967	10
4	442.26601	221.63664	I	955.59349	478.30038	9
5	513.30312	257.15520	A	842.50943	421.75835	8
6	584.34024	292.67376	A	771.47231	386.23980	7
7	683.40865	342.20796	V	700.43520	350.72124	6
8	782.47707	391.74217	V	601.36679	301.18703	5
9	897.50401	449.25564	D	502.29837	251.65282	4
10	996.57242	498.78985	V	387.27143	194.13935	3
11	1109.65649	555.33188	I	288.20302	144.60515	2
12			R	175.11895	88.06311	1

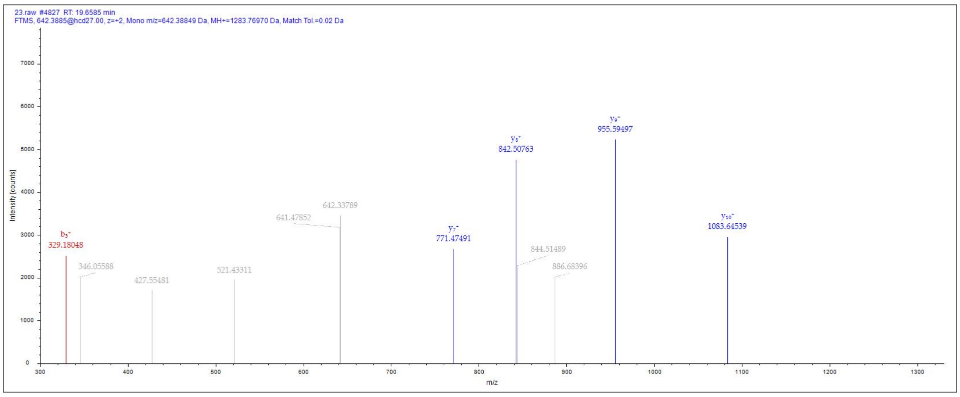

Spot 102: Haptoglobin (P00738)

Fragment matches						
**#1**	**b⁺**	**b²⁺**	**Seq.**	**y⁺**	**y²⁺**	**#2**
1	102.05496	51.53112	T			13
2	231.09755	116.05241	E	1338.61720	669.81224	12
3	288.11901	144.56314	G	1209.57461	605.29094	11
4	403.14595	202.07662	D	1152.55314	576.78021	10
5	460.16742	230.58735	G	1037.52620	519.26674	9
6	559.23583	280.12155	V	980.50474	490.75601	8
7	722.29916	361.65322	Y	881.43632	441.22180	7
8	823.34684	412.17706	T	718.37299	359.69014	6
9	936.43090	468.71909	L	617.32532	309.16630	5
10	1050.47383	525.74055	N	504.24125	252.62426	4
11	1164.51676	582.76202	N	390.19832	195.60280	3
12	1293.55935	647.28331	E	276.15540	138.58134	2
13			K	147.11280	74.06004	1

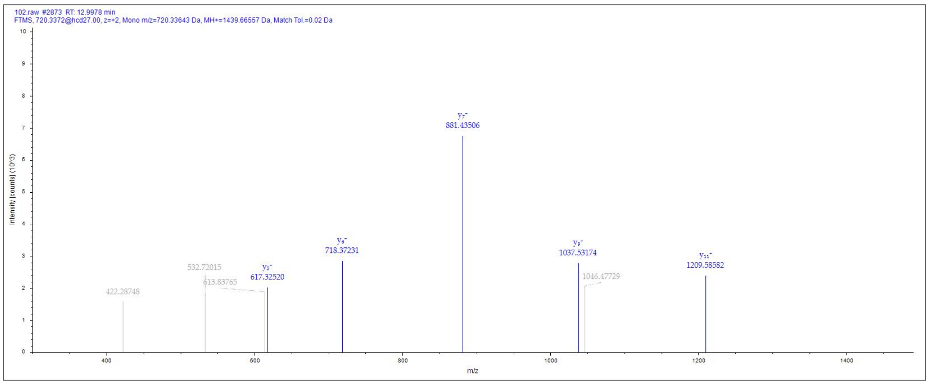

Spot 113: Haptoglobin (P00738)

Fragment matches						
**#1**	**b⁺**	**b²⁺**	**Seq.**	**y⁺**	**y²⁺**	**#2**
1	102.05496	51.53112	T			13
2	231.09755	116.05241	E	1338.61720	669.81224	12
3	288.11901	144.56314	G	1209.57461	605.29094	11
4	403.14595	202.07662	D	1152.55314	576.78021	10
5	460.16742	230.58735	G	1037.52620	519.26674	9
6	559.23583	280.12155	V	980.50474	490.75601	8
7	722.29916	361.65322	Y	881.43632	441.22180	7
8	823.34684	412.17706	T	718.37299	359.69014	6
9	936.43090	468.71909	L	617.32532	309.16630	5
10	1050.47383	525.74055	N	504.24125	252.62426	4
11	1164.51676	582.76202	N	390.19832	195.60280	3
12	1293.55935	647.28331	E	276.15540	138.58134	2
13			K	147.11280	74.06004	1

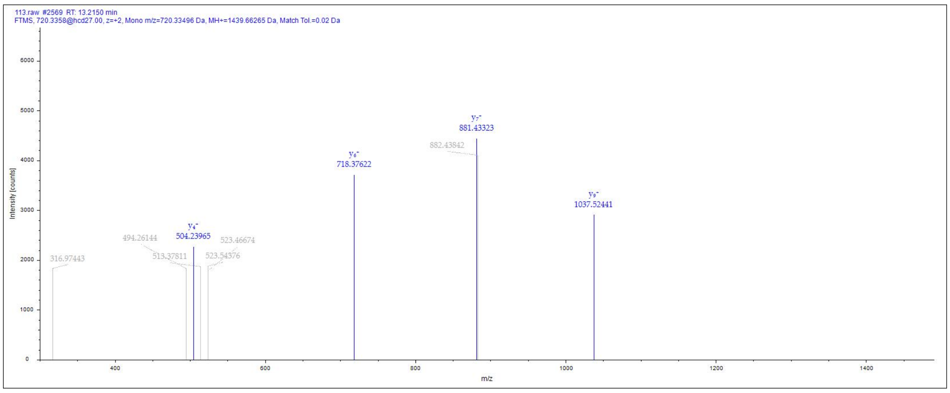

Spot 129: Haptoglobin (P00738)

Fragment matches						
#1	b⁺	b^{2+}	Seq.	y⁺	y^{2+}	#2
1	102.05496	51.53112	T			13
2	231.09755	116.05241	E	1338.61720	669.81224	12
3	288.11901	144.56314	G	1209.57461	605.29094	11
4	403.14595	202.07662	D	1152.55314	576.78021	10
5	460.16742	230.58735	G	1037.52620	519.26674	9
6	559.23583	280.12155	V	980.50474	490.75601	8
7	722.29916	361.65322	Y	881.43632	441.22180	7
8	823.34684	412.17706	T	718.37299	359.69014	6
9	936.43090	468.71909	L	617.32532	309.16630	5
10	1050.47383	525.74055	N	504.24125	252.62426	4
11	1164.51676	582.76202	N	390.19832	195.60280	3
12	1293.55935	647.28331	E	276.15540	138.58134	2
13			K	147.11280	74.06004	1

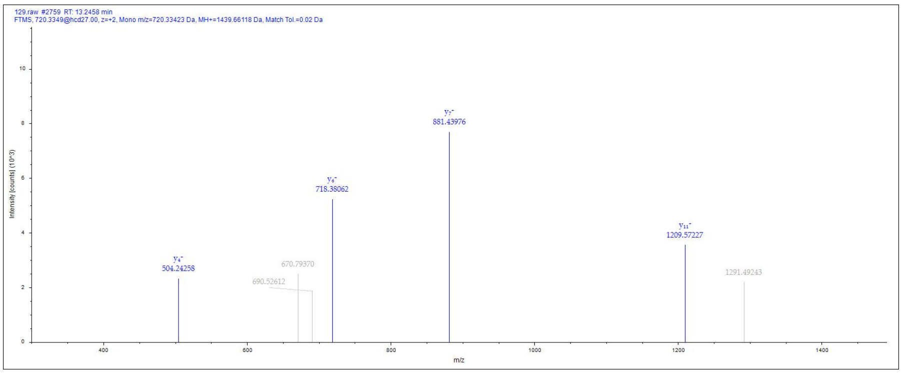

Spot 133: Ferritin light chain (P02792)

Fragment matches						
#1	b⁺	b^{2+}	Seq.	y⁺	y^{2+}	#2
1	114.09134	57.54931	L			15
2	171.11280	86.06004	G	1494.72233	747.86481	14
3	228.13427	114.57077	G	1437.70087	719.35407	13
4	325.18703	163.09715	P	1380.67941	690.84334	12
5	454.22962	227.61845	E	1283.62664	642.31696	11
6	525.26674	263.13701	A	1154.58405	577.79566	10
7	582.28820	291.64774	G	1083.54694	542.27711	9
8	695.37227	348.18977	L	1026.52547	513.76637	8
9	752.39373	376.70050	G	913.44141	457.22434	7
10	881.43632	441.22180	E	856.41994	428.71361	6
11	1044.49965	522.75346	Y	727.37735	364.19231	5
12	1157.58372	579.29550	L	564.31402	282.66065	4
13	1304.65213	652.82970	F	451.22996	226.11862	3
14	1433.69472	717.35100	E	304.16155	152.58441	2
15			R	175.11895	88.06311	1

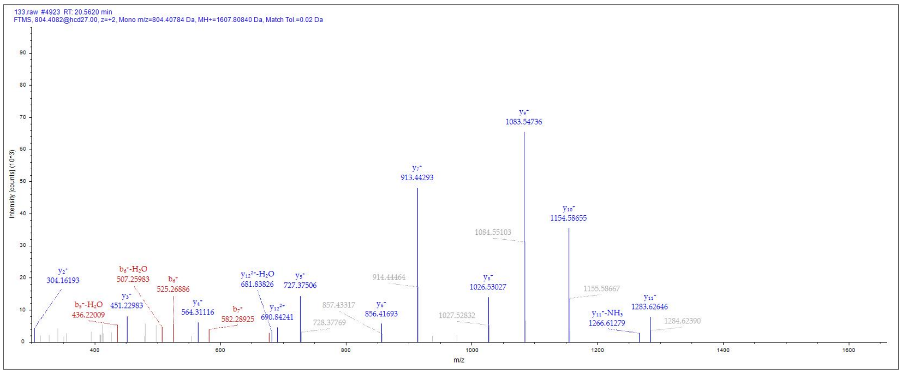

Spot 133: Hemopexin (P02790)

Fragment matches						
**#1**	**b^+**	**b^{2+}**	**Seq.**	**y^+**	**y^{2+}**	**#2**
1	115.05020	58.02874	N			11
2	262.11862	131.56295	F	1106.56292	553.78510	10
3	359.17138	180.08933	P	959.49451	480.25089	9
4	446.20341	223.60534	S	862.44174	431.72451	8
5	543.25617	272.13173	P	775.40971	388.20850	7
6	642.32459	321.66593	V	678.35695	339.68211	6
7	757.35153	379.17940	D	579.28854	290.14791	5
8	828.38864	414.69796	A	464.26159	232.63444	4
9	899.42576	450.21652	A	393.22448	197.11588	3
10	1046.49417	523.75072	F	322.18737	161.59732	2
11			R	175.11895	88.06311	1

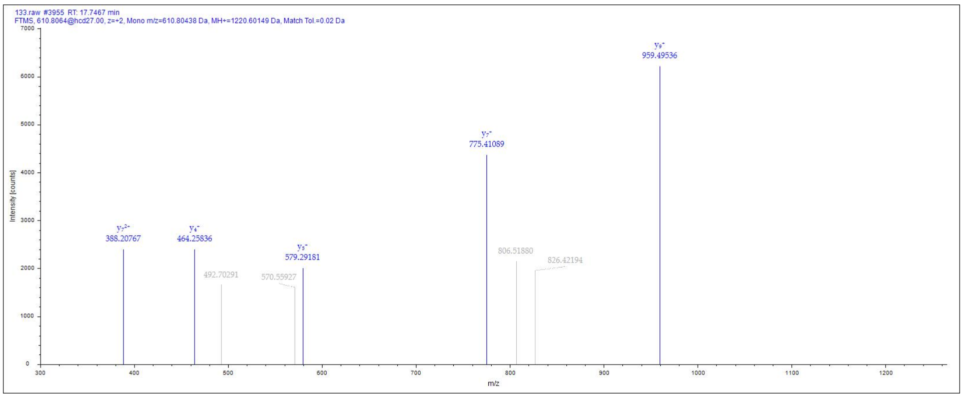

Spot 165: Beta-2-glycoprotein 1 (P02749)

Fragment matches						
**#1**	**b^+**	**b^{2+}**	**Seq.**	**y^+**	**y^{2+}**	**#2**
1	100.07569	50.54148	V			14
2	260.10634	130.55681	C-Carbamidomethyl	1403.71000	702.35864	13
3	357.15910	179.08319	P	1243.67935	622.34331	12
4	504.22752	252.61740	F	1146.62658	573.81693	11
5	575.26463	288.13595	A	999.55817	500.28272	10
6	632.28609	316.64669	G	928.52106	464.76417	9
7	745.37016	373.18872	I	871.49959	436.25343	8
8	858.45422	429.73075	L	758.41553	379.71140	7
9	987.49681	494.25205	E	645.33146	323.16937	6
10	1101.53974	551.27351	N	516.28887	258.64807	5
11	1158.56121	579.78424	G	402.24594	201.62661	4
12	1229.59832	615.30280	A	345.22448	173.11588	3
13	1328.66673	664.83701	V	274.18737	137.59732	2
14			R	175.11895	88.06311	1

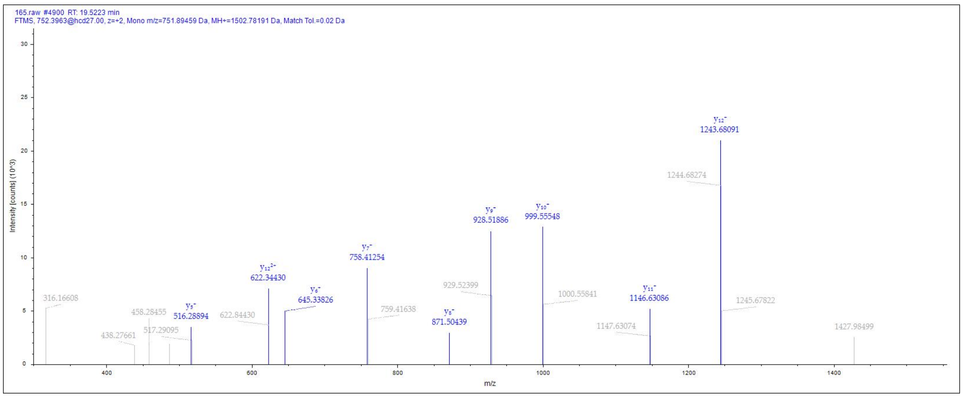

Spot 209: Complement C4-A (P0C0L4)

Fragment matches						
#1	b^+	b^{2+}	Seq.	y^+	y^{2+}	#2
1	100.07569	50.54148	V			15
2	213.15975	107.08352	L	1442.74855	721.87791	14
3	300.19178	150.59953	S	1329.66448	665.33588	13
4	413.27585	207.14156	L	1242.63246	621.81987	12
5	484.31296	242.66012	A	1129.54839	565.27783	11
6	612.37154	306.68941	Q	1058.51128	529.75928	10
7	741.41413	371.21070	E	930.45270	465.72999	9
8	869.47271	435.23999	Q	801.41011	401.20869	8
9	968.54112	484.77420	V	673.35153	337.17940	7
10	1025.56259	513.28493	G	574.28312	287.64520	6
11	1082.58405	541.79566	G	517.26165	259.13446	5
12	1169.61608	585.31168	S	460.24019	230.62373	4
13	1266.66884	633.83806	P	373.20816	187.10772	3
14	1395.71143	698.35936	E	276.15540	138.58134	2
15			K	147.11280	74.06004	1

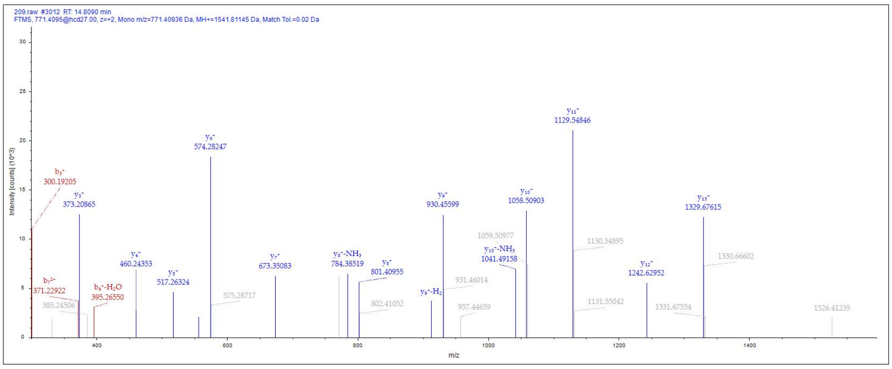

Spot 209: Complement C4-B (P0C0L5)

Fragment matches						
#1	b^+	b^{2+}	Seq.	y^+	y^{2+}	#2
1	100.07569	50.54148	V			15
2	213.15975	107.08352	L	1442.74855	721.87791	14
3	300.19178	150.59953	S	1329.66448	665.33588	13
4	413.27585	207.14156	L	1242.63246	621.81987	12
5	484.31296	242.66012	A	1129.54839	565.27783	11
6	612.37154	306.68941	Q	1058.51128	529.75928	10
7	741.41413	371.21070	E	930.45270	465.72999	9
8	869.47271	435.23999	Q	801.41011	401.20869	8
9	968.54112	484.77420	V	673.35153	337.17940	7
10	1025.56259	513.28493	G	574.28312	287.64520	6
11	1082.58405	541.79566	G	517.26165	259.13446	5
12	1169.61608	585.31168	S	460.24019	230.62373	4
13	1266.66884	633.83806	P	373.20816	187.10772	3
14	1395.71143	698.35936	E	276.15540	138.58134	2
15			K	147.11280	74.06004	1

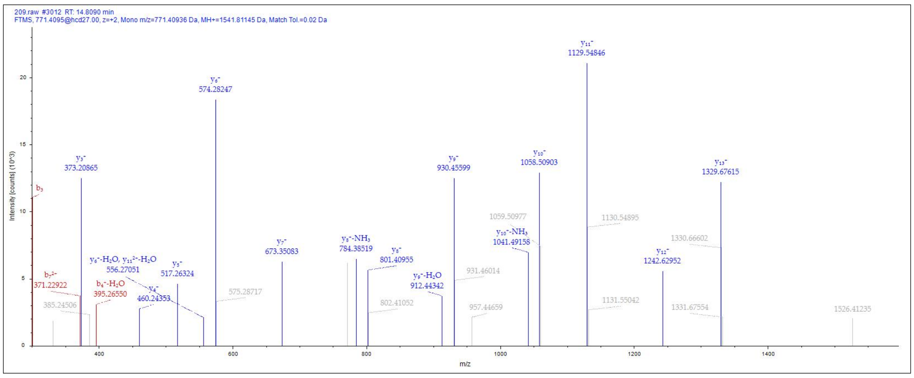

Spot 256: Prothrombin (P00734)

Fragment matches						
#1	b⁺	b²⁺	Seq.	y⁺	y²⁺	#2
1	88.03930	44.52329	S			9
2	145.06077	73.03402	G	1061.51968	531.26348	8
3	258.14483	129.57605	I	1004.49821	502.75274	7
4	387.18743	194.09735	E	891.41415	446.21071	6
5	547.21807	274.11268	C-Carbamidomethyl	762.37155	381.68942	5
6	675.27665	338.14196	Q	602.34091	301.67409	4
7	788.36072	394.68400	L	474.28233	237.64480	3
8	974.44003	487.72365	W	361.19827	181.10277	2
9			R	175.11895	88.06311	1

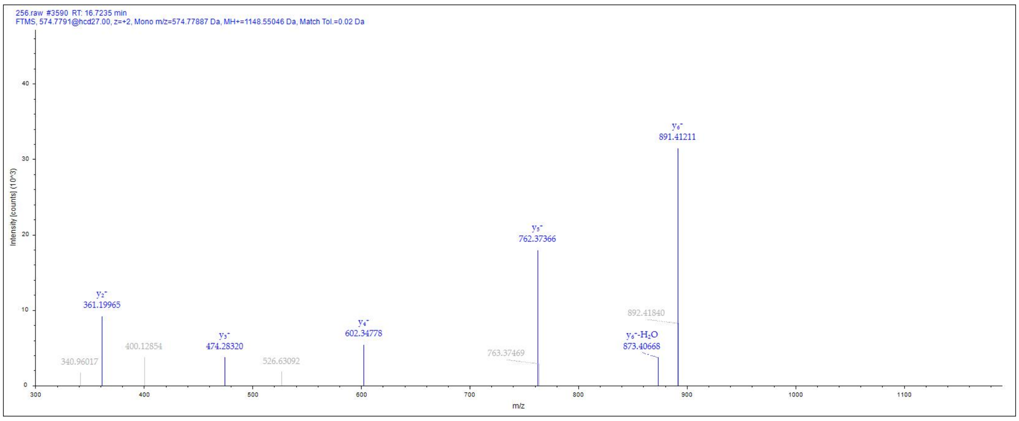

Spot 256: Insulin receptor substrate 4 (O14654)

Fragment matches						
#1	b⁺	b²⁺	Seq.	y⁺	y²⁺	#2
1	148.04268	74.52498	M-Oxidation			10
2	304.14379	152.57553	R	1153.54187	577.27457	9
3	375.18090	188.09409	A	997.44076	499.22402	8
4	488.26496	244.63612	L	926.40364	463.70546	7
5	648.29561	324.65144	C-Carbamidomethyl	813.31958	407.16343	6
6	719.33273	360.17000	A	653.28893	327.14810	5
7	834.35967	417.68347	D	582.25182	291.62955	4
8	963.40226	482.20477	E	467.22487	234.11608	3
9	1126.46559	563.73643	Y	338.18228	169.59478	2
10			R	175.11895	88.06311	1

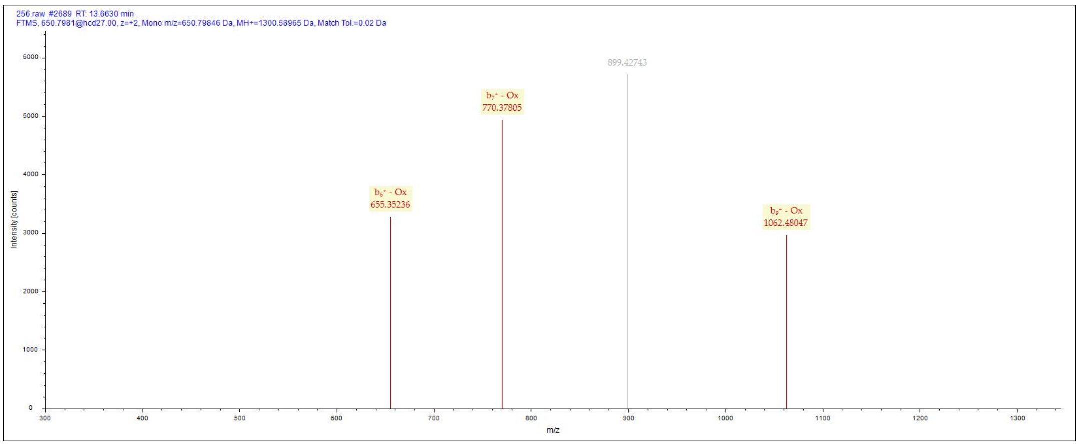

Supplemental Information: Full Blot Images of WB in the manuscript

Title

Longitudinal proteomics study of serum changes after allogeneic HSCT reveals potential markers of metabolic complications related to aGvHD

Authors

Sing Ying Wong, Seiko Kato, Frans Rodenburg, Arinobu Tojo, Nobuhiro Hayashi

Description

In the supplemental information, the original images are shown without “enhance adjustment” and increased “highlight” functions in Keynote.

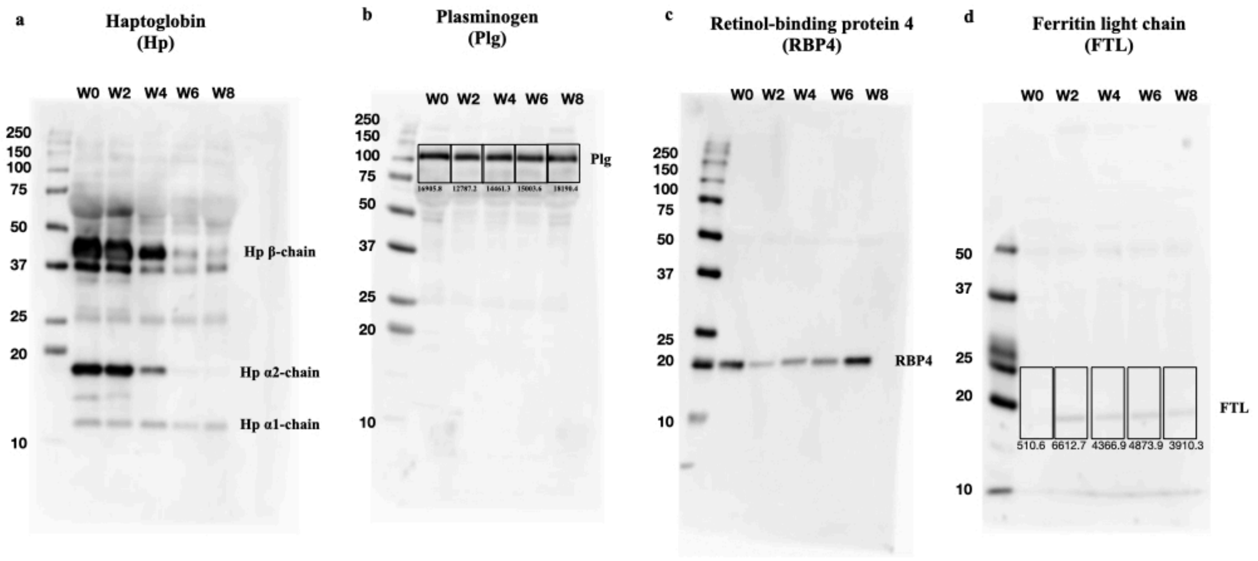

Fig. 6
